# An Alternative Approach to Detecting Cancer Cells by Multi-Directional Fluorescence Detection System Using Cost-Effective LED and Photodiode

**DOI:** 10.3390/s19102301

**Published:** 2019-05-18

**Authors:** Kyoungrae Cho, Jeong-hyeok Seo, Gyeongyong Heo, Se-woon Choe

**Affiliations:** 1 Department of Medical IT Convergence Engineering, Kumoh National Institute of Technology, Gumi 39253, Korea; jkl6464@kumoh.ac.kr (K.C.); whiteneco@yonsei.ac.kr (J.-h.S.); 2 Department of Electronic Engineering, Dong-eui University, Busan 47340, Korea; hgycap@deu.ac.kr

**Keywords:** fluorescence detection system, light-emitting diode, photodiode, cancer cells

## Abstract

The enumeration of cellular proliferation by covering from hemocytometer to flow cytometer is an important procedure in the study of cancer development. For example, hemocytometer has been popularly employed to perform manual cell counting. It is easily achieved at a low-cost, however, manual cell counting is labor-intensive and prone to error for a large number of cells. On the other hand, flow cytometer is a highly sophisticated instrument in biomedical and clinical research fields. It provides detailed physical parameters of fluorescently labeled single cells or micro-sized particles depending on the fluorescence characteristics of the target sample. Generally, optical setup to detect fluorescence uses a laser, dichroic filter, and photomultiplier tube as a light source, optical filter, and photodetector, respectively. These components are assembled to set up an instrument to measure the amount of scattering light from the target particle; however, these components are costly, bulky, and have limitations in selecting diverse fluorescence dyes. Moreover, they require multiple refined and expensive modules such as cooling or pumping systems. Thus, alternative cost-effective components have been intensively developed. In this study, a low-cost and miniaturized fluorescence detection system is proposed, i.e., costing less than 100 US dollars, which is customizable by a 3D printer and light source/filter/sensor operating at a specific wavelength using a light-emitting diode with a photodiode, which can be freely replaceable. The fluorescence detection system can quantify multi-directional scattering lights simultaneously from the fluorescently labeled cervical cancer cells. Linear regression was applied to the acquired fluorescence intensities, and excellent linear correlations (*R*^2^ > 0.9) were observed. In addition, the enumeration of the cells using hemocytometer to determine its performance accuracy was analyzed by Student’s *t*-test, and no statistically significant difference was found. Therefore, different cell concentrations are reversely calculated, and the system can provide a rapid and cost-effective alternative to commercial hemocytometer for live cell or microparticle counting.

## 1. Introduction

Since precise enumeration of cellular proliferation or differentiation is of importance during cancer development, the number of cells must be regularly accessed. To determine the number of cells, various types of equipment covering from hemocytometer to flow cytometer are available. For example, hemocytometer has been generally employed to perform manual counting of propagated cells in vitro [1]. Although it is easily achieved at low-cost, manual cell counting using hemocytometer is labor-intensive and prone to error for a large number of cells due to error-inducing conditions [2]. Moreover, it suffers from various drawbacks that impede the accuracy of hemocytometer counts, including a lack of statistical strength at low concentration, inaccurate enumeration due to device misapplication, and user-dependent variation. On the other hand, automated cell counting equipment is a valuable research technique used in cell screening and biochemical analysis to determine many unique characteristics of live microorganisms [3]. In the mid 1900s, flow cytometer was considered as a single-parameter instrument that can detect only the size of cells [4]. Since the past 70 years, flow cytometry has evolved as an essential technique used to quantify 14 parameters (cell viability, glutathione, adherence, etc.) in a single trial in various devices ranging from hematology analyzers to cutting-edge tools [5]. These multiple optical parameters were achieved based on light scattering in the physical interaction between fluorescent stained cells and the specific wavelength of the excitation light used [6,7]. 

Forward scatter (FSC) is the collected diffraction light from the cell along the path of the light source. Because FSC can be altered by cell surface, it is commonly used for immunophenotyping, which is appropriate for detecting the diameter and size of cells in a saline buffer solution by using the optical characteristics that are inversely proportional to the penetration ratio of light [8]. On the contrary, side scatter (SSC) mostly measures the reflected and refracted light, caused by the dimension of particulates and cells, in the direction perpendicular to the light source [9]. Depending on the cell type, the optical features, such as scattered light and emitted fluorescence from the target cells stained with fluorescence dye or monoclonal antibodies, are different, which play an important role [10]. This approach allows to provide precise resolution and high sensitivity while consuming low amounts of sample volume. 

Generally, fluorescence detection systems are composed of a gas laser, dichroic filter, and photomultiplier tube (PMT) used as a light source, optical filter, and photodetector, respectively. However, they have some drawbacks, such as high costs of operation and maintenance. For example, gas lasers, widely used as a light source that excites fluorescent probes, can generate powerful outputs and thus overheat. In addition, their relatively short lifespan and high cost limit their use, and a trained professional is required to operate them. Moreover, while converting electrical energy to light energy, they are highly affected by the thermal effects, which can interfere with their continuous and constant output [11]. Under these constraints, a gas laser requires an additional cooling system, which makes visual fluorescence detection system difficult to be miniaturized [11,12]. In contrast, a compact light-emitting diode (LED) may overcome these limitations by low energy consumption (0.1–2 W), long lifetime (5000–23,000 h), low cost (1–10 US dollars), and no requirement of trained professionals in its use. Moreover, spectral selectivity and output stability of LEDs make them more attractive as an alternative light source to conventional gas lasers [12,13]. Various dichroic optical filters used for high transmission rate with narrow pass bands are extensively applied to commercial fluorescence detection system; however, these filters are expensive, and users can select only a single wavelength at a time [14]. Cost-effective filter booklets have shown appropriate performance in many studies and satisfied the requirements of researchers [15,16]. PMTs have been used in many cell screening and biochemical analysis because of their ability to provide single photoelectron sensitivity and enhanced signal-to-noise ratio in a limited amount of light [17]. However, PMTs suffer from some drawbacks such as high cost, high polarizing voltage, and overheating [18]. Compared to a PMT, a photodiode (PD) is developed at low cost with high photoelectric conversion efficiency, wide spectral detecting range, and relatively high gain with low noise if it is applied with a high quality amplifier [13]. 

In this study, a low-cost and multi-directional fluorescence detection system using a cost-effective LED is proposed with a commercial optical filter booklet and a low-cost PD. The system is designed to be portable, lightweight, and easy to build using a 3D printer. The excitation and emission filters can be chosen from one of the inexpensive filter booklets; hence, users can freely select optical filters and fluorescent dyes depending on their purpose. To evaluate the feasibility of the proposed detection system, FSC and SSC fluorescence intensities from Rhodamine-6G (R6G) stained HeLa cells with several different concentrations were measured simultaneously in two different light directions. Subsequently, the fluorescence intensities were compared with the enumeration of the cells using hemocytometer to determine its performance accuracy and statistically analyzed by Student’s *t*-test. Moreover, quantified fluorescence intensities with excellent linear correlations were converted and applied to estimate the number of target cells by using a linear regression model.

## 2. Materials and Methods

**Figure 1:**
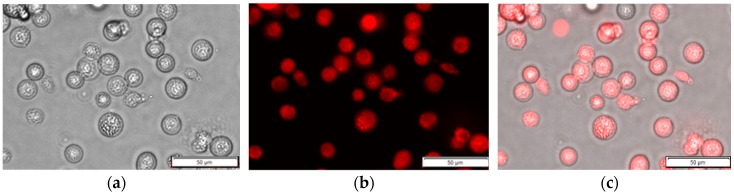
Representative R6G treated images of HeLa cells under a microscope with 200 magnification (**a**) bright field image; (**b**) fluorescence image; (**c**) merged image of fluorescently labeled HeLa cells.

HeLa cells (Korea Cell Line Bank, Korea) were prepared as described in [19]. Briefly, the cells were cultured in high-glucose Dulbecco’s modified eagle medium containing 10% fetal bovine serum with 1% penicillin–streptomycin. The prepared cells were stored in vented cell culture flasks maintained in a 37 °C incubator with 5% of CO_2_. The HeLa cells were washed three times with phosphate-buffered saline (PBS) to remove cell suspensions and protein before trypsinization. The cells were centrifuged with a relative centrifugal force of 250, which is recommended for live HeLa cells. The isolated cells were incubated in a 1 µM R6G (excitation wavelength: 528 nm, emission wavelength: 551 nm, Sigma-Aldrich, St. Louis, MO, USA) solution at 37 °C for 20 min to stain the HeLa cells. All the prepared cells were washed three times with PBS to remove the supernatant and unbound R6G. Subsequently, PBS was added up to 3 mL in a transparent glass cuvette (HE 100-600QG, HELLMA, Müllheim, Germany). Five different cell concentrations were prepared ranging from 1 × 10^6^ to 5 × 10^6^ cells with an increment of 1 × 10^6^ cells. HeLa cells treated and non-treated with R6G were assigned to an experimental group (number of samples = 12) and control group (number of samples = 12), respectively, at a certain concentration and measured to compare their fluorescence intensities. Representative R6G treated images of HeLa cell under fluorescence microscope with 200 magnification were shown in Figure 1. 

**Figure 2:**
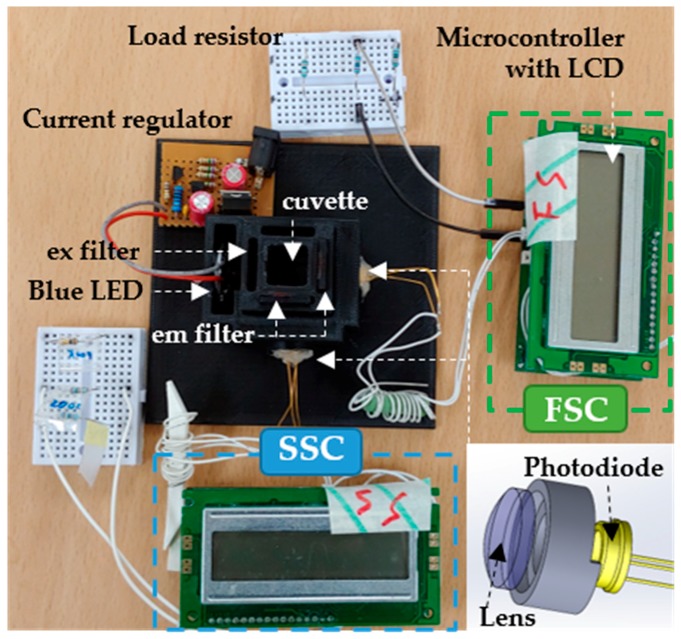
Proposed fluorescence detection system. Light-emitting diode (LED) with specific wavelength excites the HeLa cells sample through the excitation filter (ex filter). Emission light including side scatter (SSC) and forward scatter (FSC) is collected through the emission filter (em filter). Collected light is analyzed and displayed on the liquid crystal display (LCD) module.

The proposed fluorescence detection system is shown in Figure 2. To power a 470 nm LED (Photron, 3 W, Tokyo, Japan), an output current driven from a 360 mA current regulator is directly connected to the LED driver, which makes the LED generate a 1.4 W power. To minimize the thermal effects, the LED was warmed for 30 min before the test, and the temperature was controlled to be less than 25 °C. Light from the LED excites the fluorescently treated HeLa cells through an excitation filter. The scattered lights (SSC and FSC) passing through the emission filter in two different directions were collected by a PD (FDS100, 350–1100 nm, Thorlabs, Newton, NJ, USA) in the module. To augment the collection of the scattered lights, a plano-convex lens (BK7, Plano-Convex Lens, Thorlabs, Newton, NJ, USA) was installed in front of the PD based on its focal depth. Optical filters (filter booklets, Edmund Optics, Barrington, NJ, USA) for filtering excitation and emission wavelengths were freely selected and easily installed into the customized empty slots in this module based on the selected fluorescence dye.

**Figure 3:**
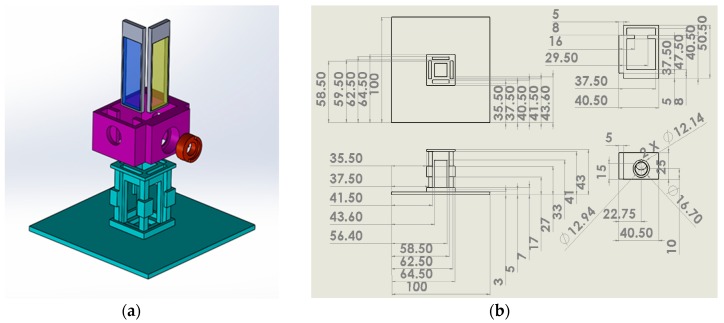
Computer aided design (CAD) of the main module: (**a**) top 3D view including filter holder (gray region), LED & lens holder (purple region), cuvette holder (green region), and lens and PD holder (red region); (**b**) isometric view of cuvette holder, lens, and PD holder parts.

As shown in Figure 3, the cuvette holder was designed using a 3D computer aided design program and subsequently printed using a 3D printer (3DP-310F, Cubicon Inc., Seongnam, Gyeonggido, Korea) with a 100% acrylonitrile butadiene styrene (ABS) copolymer. The holder was aimed to be compact in size and simple in structure, and to block the unnecessary lights that can cause noise within the system, all the hardware devices were printed using black ABS. There were four empty slots for filter sets in two different directions, parallel and perpendicular to the light pathway. Irradiation direction from the LED could be controlled by blocking the unnecessary slots except the pathway to the PD.

**Figure 4:**
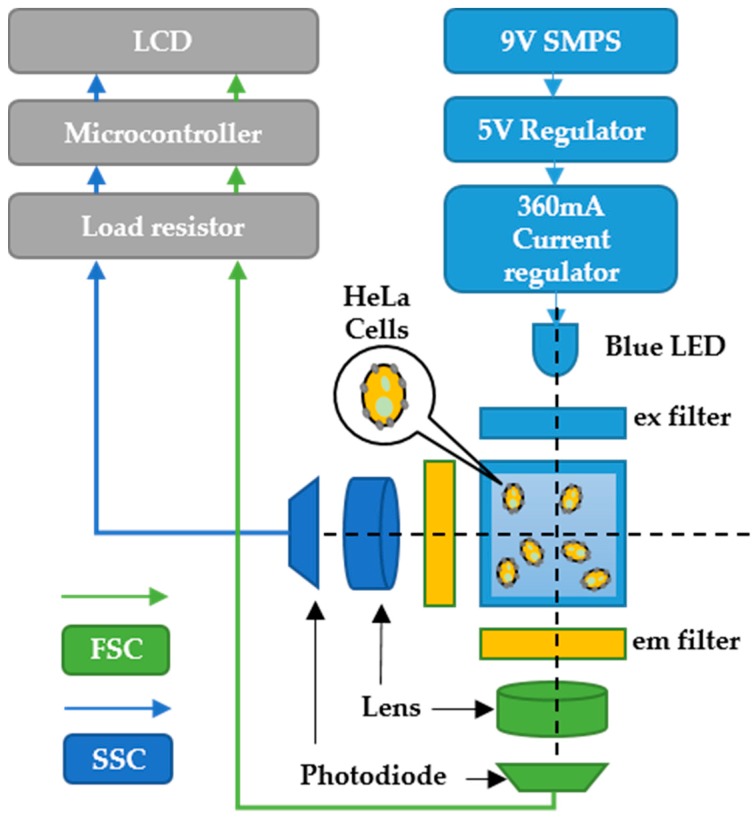
Schematic design of the fluorescence detection system.

The stained cells were filled up to 3 mL in PBS with specific cell concentrations in a glass cuvette and irradiated in the dark for 5 min at ambient temperature to measure the FSC and SSC. For example, as in Figure 4, the R6G stained HeLa cells were excited through one of the excitation filters among Tahitian Blue (#369, 470 nm) or Night Blue (#74, 480 nm) of a commercial filter booklet. The SSC and FSC from the cells were filtered using Chromo Green (#389, 500 nm) filter and focused towards the PD using a plano-convex lens. Similar to the LED operation, the PD was also tuned for 30 min before the test to suppress the thermal effects. The measured photocurrent is digitized and converted by a low-cost microcontroller (PIC16F877A, Microchip, Chandler, AZ, USA) and quantized on an LCD in the display module. In addition, one of the three different load resistors (100, 200, and 390 kΩ) was connected to optimize and amplify the photocurrents to read.

**Figure 5:**
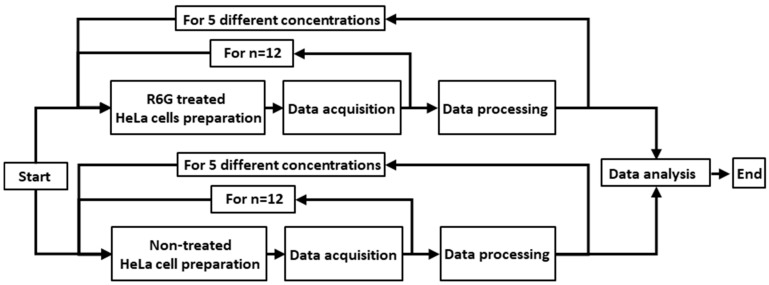
Data acquisition procedure of fluorescence detection system for a single filter set and load resistor.

The experimental procedure for the acquisition of FSC and SSC is shown in Figure 5. R6G treated (n = 12) and non-treated HeLa cells (n = 12) with five different cell concentrations ranging from 1 × 10^6^ to 5 × 10^6^ cells with an increment of 1 × 10^6^ cells were prepared to eliminate the scattering lights coming from the R6G non-treated HeLa cells. The R6G treated HeLa cells were irradiated through either of the excitation filters (no filter, #369 with 470 nm or #74 with 480 nm) by the power LED (470 nm, 3 W). Subsequently, SSCs and FSCs from the cells emitted through the emission filters (#389 with 500 nm) were collected simultaneously by one of the PD with different load resistors (100, 200, and 390 kΩ). The individually measured FSCs and SSCs of the R6G non-treated HeLa cells at a specific concentration were deducted from the treated HeLa cells with the same concentration to derive unmixed FSCs and SSCs. The trend lines and slopes of each group of the FSCs and SSCs were calculated using linear regression analysis. To evaluate the effectiveness and stability of the system, the relative standard deviation (RSD) is calculated as in Equation (1).

(1) $$\mathrm{RSD}\;\left(\%\right)=\frac{standard\;deviation\left(S\right)}{mean\left(S\right)}\times 100$$

## 3. Results and Discussion

The total cost of all the components required to be assembled and design the proposed system was under 100 US dollars, which is much less than that of a commercial fluorescence detection system [20]. Except for the 9 V switched-mode power supply (SMPS), all the electronic circuit boards were developed cost-effectively in our laboratory using commercial electronic components. The unit price of the fluorescence detection system is summarized in Table 1.

**Figure 6:**
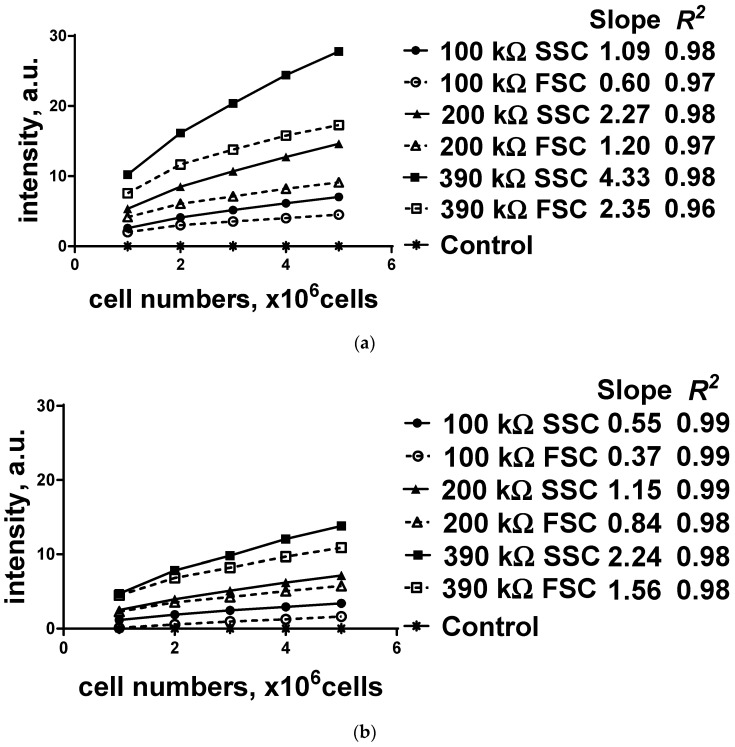

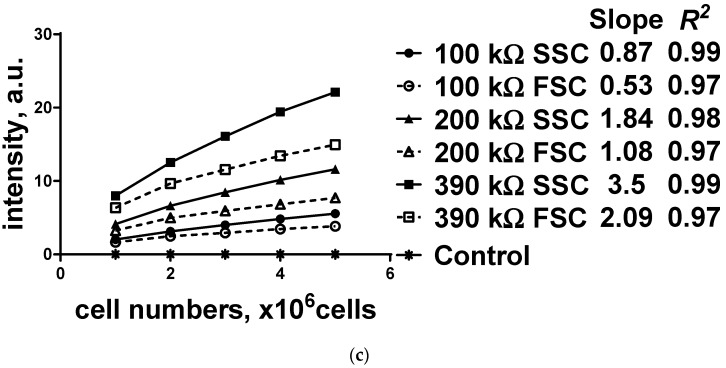
Comparison of the SSCs and FSCs of the fluorescence intensities of HeLa cells using one of the three different load resistors (100, 200, and 390 kΩ) with a specific filter set: (**a**) no excitation filter, but only emission filter (center wavelength (CWL) at 500 nm); (**b**) excitation filter of CWL at 480 nm with emission filter of CWL at 500 nm; (**c**) excitation filter of CWL at 470 nm with emission filter of CWL at 500 nm. RSD (%) was calculated for each concentration group of stained HeLa cells with one of the selected load resistors.

The unstained (control group) and stained (experimental group) HeLa cell concentrations were modified from 1 × 10^6^ to 5 × 10^6^ cells with an increment of 1 × 10^6^ cells, and the prepared sample was put into the cuvette holder in the main module. Subsequently, the FSCs and SSCs from both groups were collected using the proposed system, and the quantified values of the FSCs and SSCs from the control group were subtracted from the measured parameters of the experimental group with the same concentration to eliminate the optical interference. To amplify the photocurrents of each group, one of load resistors (100, 200, and 390 kΩ) was connected to a display module, and the results are shown in Figure 6. 

Overall, it is evident that the SSC values are greater than the FSC values, and the values are more distinguishable when a high load resistor is connected. Because the FSCs of the cells are collected in a direction parallel to the LED based on the Beer–Lambert law, they contain stronger scattering lights that must be subtracted from the measured fluorescence intensities compared to SSCs; hence, the calculated SSCs are much higher than the FSCs. In addition, the calculated fluorescence light intensities and the slope of all the experimental groups are consistently proportional to the size of the load resistor. Particularly, the slope of the high load resistors (390 and 200 kΩ) connected to the PD is double relative to that of the low load resistors (200 and 100 kΩ). All the FSCs and SSCs from the stained HeLa cells show high linearity (*R*^2^ > 0.9) proportional to cell concentrations, while the control group did not show any optical changes. Because the maximum value of RSD is less than 3%, the proposed system is considered stable for performing fluorescence detection and cell population calculation. Additionally, high current-to-voltage amplification with a high load resistor demonstrates a high slope of the trend line; hence, the proposed system can easily categorize the relative cell concentration range. Because SSCs are more delicate to display the measured fluorescence intensities in the same experimental conditions than FSCs, SSCs can be reversely applicable to calculate or estimate the cell concentration. The fluorescence intensities were digitized by the microcontroller within an ADC’s converting range, which provided readable percentage values. 

The measured SSCs and FSCs of the R6G stained HeLa cells at different concentrations are summarized in Table 2, Table 3 and Table 4. Each experiment was performed with one of the five different cell concentrations (1 × 10^6^ to 5 × 10^6^ cells), one of the three load resistors (100, 200, and 390 kΩ), and one of the optical filter sets at different center wavelength values (470, 480, and 500 nm) as excitation and emission filters. To evaluate the feasibility, sensitivity, and stability of the proposed system, quantitative values (slope, *R*^2^, and RSD (%)) were calculated. The slope of the FSCs and SSCs is proportional to the cell concentration. Particularly, the slope of a higher load resistor connected to the PD is double than that of a lower one. All scattering values from the stained HeLa cells show high linearity (*R*^2^ > 0.9) proportional to cell concentration. Because the maximum value of RSD is less than 3%, the proposed system is considered stable for performing fluorescence detection, and the measured scattering values are applied to cell population calculation. RSDs were slightly increased when the system used high load resistors; however, this can be optimized by adding a constant current module. 

**Figure 7:**
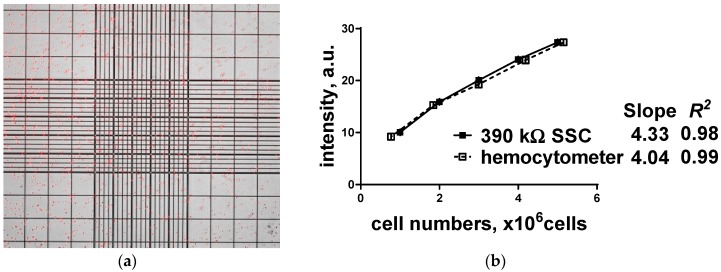
Representative R6G treated images of HeLa cells on the ruled hemocytometer chamber under a fluorescence microscope and its performance results: (**a**) merged image of bright field image and fluorescently labeled HeLa cells; (**b**) fluorescence intensity of HeLa cells using 390 kΩ load resistor with no excitation filter, but only emission filter (center wavelength (CWL) at 500 nm).

Subsequently, R6G stained HeLa cells at one of the five different cell concentrations (1 × 10^6^ to 5 × 10^6^ cells) were put into the ruled hemocytometer chamber under a fluorescence microscope, and number of cells were determined manually as in Figure 7. To evaluate the feasibility, sensitivity, and stability, quantitative values (slope, *R*^2^, and *p* value) of the five different cell concentrations (n = 10 per concentration) using hemocytometer were compared with the proposed fluorescence detection system with a specific filter set (no excitation filter, but only emission filter (center wavelength (CWL) at 500 nm). All the acquired fluorescence intensities from the stained HeLa cells show high linearity (*R*^2^ > 0.98) proportional to cell concentration. In addition, no statistically significant difference (*p* > 0.05) in the cell concentration was found between two measurement methods. All samples examined the relationship between the fluorescence intensities and the cell concentrations can be generalized as in Equation (2).

(2) $$\text{Cell number}=\left(0.005x^{2}+0.047x\right)\times 10^{6},\;\mathrm{when}\;x=\text{acquired fluorescence intensity}$$

## 4. Conclusions

A cost-effective miniaturized fluorescence detection system was proposed, which is portable, customizable by a 3D printer, and has a freely replaceable light source/filter/detector operating at a specific wavelength using a cost-effective LED, a commercially available microcontroller, and an optical filter booklet. This system can be constructed for less than 100 US dollars, and can provide an alternative way to determine the number of fluorescently labeled cells or micro-sized particles. Quantification of the targeted cancer cell concentration has been a widely used physiological parameter in cell screening analysis, and it can be measured cost-effectively using the proposed system. The system can quantify multi-directional scattering lights simultaneously from the fluorescently labeled cervical cancer cells. The results demonstrated excellent linearity (*R*^2^ > 0.9) and were confirmed by the low RSD values (less than 3%) with different R6G stained HeLa cell concentrations. A load resistor connected to a PD enables fluorescence detection to be performed precisely to distinguish the cell concentration. Moreover, it was confirmed that SSCs are more resourceful in collecting the fluorescence detection information than FSCs. Because different cell concentrations are used to calculate SSCs and FSCs, the system can provide the number of fluorescently labeled microparticles in two different directions simultaneously. The results from the proposed fluorescence detection system were compared with enumeration with hemocytometer, and no statistically significant difference was found (*p* > 0.05). Hemocytometer-based cell counting that is time-consuming and labor-intensive process requires imaging system with an appropriate magnifying objective to enumerate the concentration of the sample. In addition, a diluted sample causes errors under error-inducing conditions due to cells, remains, and debris on the grid lines, and its aggregation behavior. The proposed system does not need to prepare dilute sample solutions and provides fast enumeration for relatively a large number of cells. It may be applied to distinguish multiple types of targeted cells among randomly mixed cells in vitro using a cell-type-specific marker and provide quantitative parameters similar to hemocytometer. In this system, components such as light source and optical filter set at specific wavelengths can be easily modified; hence, users can freely select the type of the cells or fluorescence dye without requiring the presence of trained professionals.

## Figures and Tables

**Table 1:** List of components unit price to assemble the proposed fluorescence detection system.

Parts	Unit Price ($)
Microcontroller (PIC16F877A)	8
Character LCD display	6
PCB	16
Roscolux Filter Booklet (Edmund Optics)	15
Photodiode (FDS100)	14
SMPS	2
Constant current module	5
Half convex lens(N-BK7)	24
LED (Photron 3 W)	4
Total amount	94

**Table 2:** Summary of the measured SSCs and FSCs of R6G labeled HeLa cells using load resistors at different concentrations with no excitation filter and #389 emission filter (500 nm), n = 12/cell numbers.

	100 kΩ SSC (%)	100 kΩ FSC (%)	200 kΩ SSC (%)	200 kΩ FSC (%)	390 kΩ SSC (%)	390 kΩ FSC (%)
Slope	1.09	0.60	2.27	1.20	4.33	2.35
*R* ^2^	0.98	0.97	0.98	0.97	0.98	0.96
RSD (%)	0.35	0.42	0.80	0.95	2.47	1.83

**Table 3:** Summary of the measured SSCs and RSCs of R6G labeled HeLa cells using load resistors at different concentrations with #74 excitation filter (480 nm) and #389 emission filter (500 nm), n = 12/cell numbers.

	100 kΩ SSC (%)	100 kΩ FSC (%)	200 kΩ SSC (%)	200 kΩ FSC (%)	390 kΩ SSC (%)	390 kΩ FSC (%)
Slope	0.55	0.37	1.15	0.84	2.24	1.56
*R* ^2^	0.99	0.99	0.99	0.98	0.98	0.98
RSD (%)	0.21	0.21	0.44	0.41	0.91	0.94

**Table 4:** Summary of the measured SSCs and RSCs of R6G labeled HeLa cells using load resistors at different concentrations with #369 excitation filter (470 nm) and #389 emission filter (500 nm), n = 12/cell numbers.

	100 kΩ SSC (%)	100 kΩ FSC (%)	200 kΩ SSC (%)	200 kΩ FSC (%)	390 kΩ SSC (%)	390 kΩ FSC (%)
Slope	0.87	0.53	1.84	1.08	3.5	2.09
*R* ^2^	0.99	0.97	0.98	0.97	0.99	0.97
RSD (%)	0.26	0.37	0.63	0.73	1.66	1.50
